# Supplementary material for: iSCNT embryo culture system for restoration of Cervus nippon hortulorum, presumed to be sika deer in the Korean Peninsula
Source: PLoS One. 2024 Apr 18;19(4):e0300754. doi: 10.1371/journal.pone.0300754 (PMC11025863; doi:10.1371/journal.pone.0300754)
Supplement: S1 File — (DOCX) [file pone.0300754.s001.docx]

**Table legends**

**Table 1. Differences in the mt-DNA sequence of each sika deer sample**

|  | Mitochondria DNA D-Loop Sequencing | |  | |
| --- | --- | --- | --- | --- |
| CnD22166  CnD22119  CnD11102  CnDJ8  CnDJ6  WCnD | --CACCTCCCTAGACTCAGGGAAGAAGCCATAGCCCCACTATCAACACCCAAAGCTGAAGTTCTATTTAAACTATTCCCTGACGC  AACACCTCCCTAGACTCA-GGAAGAAGCCATAGCCCCACTATCAACACCCAAAGCTGAAGTTCTATTTAAACTATTCCCTGACGC  --CACCT-CCTAGACTCA-GGAAGAAGCCATAGCCCCACTATCAACACCCAAAGCTGAAGTTCTATTTAAACTATTCCCTGACGC  --CACCT-CCTAGACTCAGGGAAGAAGCCATAGCCCCACTATCAACACCCAAAGCTGAAGTTCTATTTAAACTATTCCCTGACGC  --CACCTCCCTAGACTCAGGGAAGAAGCCATAGCCCCACTATCAACACCCAAAGCTGAAGTTCTATTTAAACTATTCCCTGACGC  AACACCT-CCTAGACTCAGGGAAGAAGCCATAGCCCCACTATCAACACCCAAAGCTGAAGTTCTATTTAAACTATTCCCTGACGC | TTATTAATATAGTTCCATAAAAATCAAGAACTTTATCAGTATTAAATTTCCAAAAAATTTTAATATTTTAATACAGCTTTCTACT  TTATTAATATAGTTCCATAAAAATCAAGAACTTTATCAGTATTAAATTTCC-AAAAATTTTAATATTTTAATACAGCTTTCTACT  TTATTAATATAGTTCCATAAAAATCAAGAACTTTATCAGTATTAAATTTCCAAAAAATTTTAATATTTTAATACAGCTTTCTACT  TTATTAATATAGTTCCATAAAAATCAAGAACTTTATCAGTATTAAATTTCC-AAAAATTTTAATATTTTAATACAGCTTTCTACT  TTATTAATATAGTTCCATAAAAATCAAGAACTTTATCAGTATTAAATTTCC-AAAAATTTTAATATTTTAATACAGCTTTCTACT  TTATTAATATAGTTCCATAAAAATCAAGAACTTTATCAGTATTAAATTTCCAAAAAATTTTAATATTTTAATACAGCTTTCTACT | 172 |  |
| 173 | CAACATCCAATTTACATTTTATGTCCTACTAATTACACAACAAAGCACGTGATATAACCTTATGTGTTTGTAGTACATAAAATTA  CAACATCCAATTTACATTTTATGTCCTACTAATTACACAGCAAAACACGTGGTATAACCTTATATGTTTGTAGTACATAAAACTA  CAACATCCAATTTACATTTTATGTCCTACTAATTACACAACAAAGCACGTGATATAACCTTATGTGTTTGTAGTACATAAAATTA  CAACATCCAATTTACATTTTATGTCCTACTAATTACACAGCAAAACACGTGGTATAACCTTATATGTTTGTAGTACATAAAACTA  CAACATCCAATTTACATTTTATGTCCTACTAATTACACAGCAAAACACGTGGTATAACCTTATATGTTTGTAGTACATAAAACTA  CAACATCCAATTTACATTTTATGTCCTACTAATTACACAGCAAAACACGTGATACAACCTTATGTGTTTGTAATACATAAAATTA | ATGCATTAAGGCACACATGTACAATGGTACATAAAATCGGTGTATAGGACATATTATGCATAATAGTACATAAATTAATGTATTA  GTGCATTAAGACACAC--GTATAATAGCACATAAAATCGGTGTATAGGACATATTATGTATAATAGTACATAAATTAATGTATCA  ATGCATTAAGGCACACATGTACAATGGTACATAAAATCGGTGTATAGGACATATTATGCATAATAGTACATAAATTAATGTATTA  GTGCATTAAGACACAC--GTATAATAGCACATAAAATCGGTGTATAGGACATATTATGTATAATAGTACATAAATTAATGTATCA  GTGCATTAAGACACAC--GTATAATAGCACATAAAATCGGTGTATAGGACATATTATGTATAATAGTACATAAATTAATGTATCA  GTGCATTAAGACACACATGTATAACAGTACATAAAATCGGTGTATAGGACATATTATGTATAATAGTACATAAATTAATGTATTA | 344 |  |
| 345 | GGACATACTATGTATAATAGTACATTATATTATATGCCCCATGCTTATAAGCATGTATTTTCTATTATTTACAGTACATAGTACA  GGACATATTATGTATAATAGTACATTATATTATATGCCCCATGCTTATAAGCATGTATTTTCTATTATCTACAGTACATAGTACA  GGACATACTATGTATAATAGTACATTATATTATATGCCCCATGCTTATAAGCATGTATTTTCTATTATTTACAGTACATAGTACA  GGACATATTATGTATAATAGTACATTATATTATATGCCCCATGCTTATAAGCATGTATTTTCTATTATCTACAGTACATAGTACA  GGACATATTATGTATAATAGTACATTATATTATATGCCCCATGCTTATAAGCATGTATTTTCTATTATCTACAGTACATAGTACA  GGACATATTATGTATAATAGTACATTATATTATATGCCCCATGCTTATAAGCATGTATTTTCTATCATTTATAGTACATAGTACA | TAATGTTGCTTATCGTACATAGCACATTGAGTCAAATCAGTCCTCGTCAACATGCGTATCCCGTCCACTAGATCACGAGCTTGAT  TAATGTTGTTCATCGTACATAGTGCATTAAGTCAAATCAGTCCTCGTCAGCATGCGTATCCCGTCCCCTAGATCACGAGCTTAAT  TAATGTTGCTTATCGTACATAGCACATTGAGTCAAATCAGTCCTCGTCAACATGCGTATCCCGTCCACTAGATCACGAGCTTGAT  TAATGTTGTTCATCGTACATAGTGCATTAAGTCAAATCAGTCCTCGTCAGCATGCGTATCCCGTCCCCTAGATCACGAGCTTAAT  TAATGTTGTTCATCGTACATAGTGCATTAAGTCAAATCAGTCCTCGTCAGCATGCGTATCCCGTCCCCTAGATCACGAGCTTAAT  TGATGTTGTCCATCGTACATAGTGCATCAAGTCAAATCAGTCCTCGTCAACATGCGTATCCCGTCCCCTAGATCACGAGCTTGAT | 516 |  |
| 517 | CACCATGCCGCGTGAAACCAGCAACCCGCTGGGCAGGGATCCCTCTTCTCGCTCCGGGCCCATGAATTGTGGGGGTAGCTATTTA  CACCATGCCGCGTGAAACCAACAACCCGCTGGGCAGGGATCCCTCTTCTCGCTCCGGGCCCATGAATCGTGGGGGTAGCTATTTA  CACCATGCCGCGTGAAACCAGCAACCCGCTGGGCAGGGATCCCTCTTCTCGCTCCGGGCCCATGAATTGTGGGGGTAGCTATTTA  CACCATGCCGCGTGAAACCAACAACCCGCTGGGCAGGGATCCCTCTTCTCGCTCCGGGCCCATGAATCGTGGGGGTAGCTATTTA  CACCATGCCGCGTGAAACCAACAACCCGCTGGGCAGGGATCCCTCTTCTCGCTCCGGGCCCATGAATCGTGGGGGTAGCTATTTA  CACCATGCCGCGTGAAACCAGCAACCCGCTGGGCAGGGATCCCTCTTCTCGCTCCGGGCCCATGAATTGTGGGGGTAGCTATTTA | ATGAACTTTATCAGACATCTGGTTCTTTTTTCAGGGCCATCTCACCTAAAATCGCCCACTCCTTGTAATATAAGACATCTCGATG  ATGAACTTTATCAGACATCTGGTTCTTTTTTCAGGGCCATCTCACCTAAAATCGCCCACTCCTTGCAATATAAGACATCTCGATG  ATGAACTTTATCAGACATCTGGTTCTTTTTTCAGGGCCATCTCACCTAAAATCGCCCACTCCTTGTAATATAAGACATCTCGATG  ATGAACTTTATCAGACATCTGGTTCTTTTTTCAGGGCCATCTCACCTAAAATCGCCCACTCCTTGCAATATAAGACATCTCGATG  ATGAACTTTATCAGACATCTGGTTCTTTTTTCAGGGCCATCTCACCTAAAATCGCCCACTCCTTGCAATATAAGACATCTCGATG  ATGAATTTTATCAGACATCTGGTTCTTTTTTCAGGGCCATCTCACCTAAAATCGCCCACTCCTTGCAATATAAGACATCTCGATG | 688 |  |
| 689 | GACTAATGACTAATCAGCCCATGCTCACACATAACTGTGGTGTCATACATTTGGTATTTTTAATTTTTGGGGGGATGCTTGGACT  GACTAATGACTAATCAGCCCATGCTCACACATAACTGTGGTGTCATACATTTGGTATTTTTAATTTTTGGGGGGATGCTTGGACT  GACTAATGACTAATCAGCCCATGCTCACACATAACTGTGGTGTCATACATTTGGTATTTTTAATTTTTGGGGGGATGCTTGGACT  GACTAATGACTAATCAGCCCATGCTCACACATAACTGTGGTGTCATACATTTGGTATTTTTAATTTTTGGGGGGATGCTTGGACT  GACTAATGACTAATCAGCCCATGCTCACACATAACTGTGGTGTCATACATTTGGTATTTTTAATTTTTGGGGGGATGCTTGGACT  GACTAATGACTAATCAGCCCATGCTCACACATAACTGTGGTGTCATACATTTGGTATTTTTAATTTTTGGGGGGATGCTTGGACT | CAGCAATGGCCGTCTGAGGCCCCGTCCCGGAGCATGAATTGTAGCTGGACTTAACTGCATCTTGAGCATCCCCATAATGGTAGGC  CAGCAATGGCCGTCTGAGGCCCCGTCCCGGAGCATAAATTGTAGCTGGACTTAACTGCATCTTGAGCATCCCCATAATGGTAGGC  CAGCAATGGCCGTCTGAGGCCCCGTCCCGGAGCATGAATTGTAGCTGGACTTAACTGCATCTTGAGCATCCCCATAATGGTAGGC  CAGCAATGGCCGTCTGAGGCCCCGTCCCGGAGCATAAATTGTAGCTGGACTTAACTGCATCTTGAGCATCCCCATAATGGTAGGC  CAGCAATGGCCGTCTGAGGCCCCGTCCCGGAGCATAAATTGTAGCTGGACTTAACTGCATCTTGAGCATCCCCATAATGGTAGGC  CAGCAATGGCCGTCTGAGGCCCCGTCTCGGAGCATGAATTGTAGCTGGACTTAACTGCATCTTGAGCATCCCCATAATGGTAGGC | 860 |  |
| 861 | GCAGGGCATTGCAGTCAATGGTCACAGGACATAGTTATTATTTCATGAATCAACCCTAAGATCTATTTCCCCCCCCTTCTTATTT  GCAGGGCATTACAGTCAATGGTCACAGGACATAGTTATTATTCCATGAATCAACCCTAAGATCTATTTCCCCCCCCTCCTTA-TT  GCAGGGCATTGCAGTCAATGGTCACAGGACATAGTTATTATTTCATGAATCANCCCTAAGATCTATTTCCCCCCCCTTCTTATTT  GCAGGGCATTACAGTCAATGGTCACAGGACATAGTTATTATTCCATGAATCAACCCTAAGATCTATTTCCCCCCCCTTCTTA-TT  GCAGGGCATTACAGTCAATGGTCACAGGACATAGTTATTATTCCATGAATCAACCCTAAGATCTATTTCCCCCCCCTTCTTA-TT  GCAGGGCATTGCAGTCAATGGTCACAGGACATAGTTATTATTCCATGAATCAACCCTAAGATCTATTTCCCCCCCCTTCTTATTT | TTTCCCCCTTATATAGTTATCACCTTTTTTAACACACTTTTCCCTAGATATTATTTTAAATTTATCACATTTCCAATACTCAAAT  TTTCCCCCTTATATAGTTATCACCTTTTTTAACACACTTTTCCCTAGATATTATTTCAAATTTATCACATTTCCAATACTCAAAT  TTTCCCCCTTATATAGTTATCACCTTTTTTAACACACTTTTCCCTAGATATTATTTTAAATTTATCACATTTCCAATACTCAAAT  TTTCCCCCTTATATAGTTATCACCTTTTTTAACACACTTTTCCCTAGATATTATTTCAAATTTATCACATTTCCAATACTCAAAT  TTTCCCCCTTATATAGTTATCACCTTTTTTAACACACTTTTCCCTAGATATTATTTCAAATTTATCACATTTCCAATACTCAAAT  TTTCCCCCTTATATAGTTATCACCTTTTTTAACACACTTTTCCCTAGATATTATTTTAAATTTATCACATTTCCAATACTCAAAT | 1032 |  |
| 1033 | TAGCACTCCAGAGGGAGGTAAGTATATAAACGCCAATTTTTCCCTAATT-ACGCATAGTTAATGTAGCTTAAACAACAAAGCAAG  TGGCACTCCAGAGGGAGGTAAGTATATAAACGCCAATTTTTCCCTAATT-GCGCATAGTTAATGTAGCTTAAACAGCAAAGCAAG  TAGCACTCCAGAGGGAGGTAAGTATATAAACGCCAATTTTTCCCTAATT-ACGCATAGTTAATGTAGCTTAAACAACAAAGCAAG  TGGCACTCCAGAGGGAGGTAAGTATATAAACGCCAATTTTTCCCTAATT-GCGCATAGTTAATGTAGCTTAAACAGCAAAGCAAG  TGGCACTCCAGAGGGAGGTAAGTATATAAACGCCAATTTTTCCCTAATT-GCGCATAGTTAATGTAGCTTAAACAGCAAAGCAAG  TAGCACTCCAGAGGGAGGTAAGTATATAAACGCCAATTTTTCCCTAATTAGCGCATAGTTAATGTAGCTTAAACAGCAAAG---- | GCA-CTGAAAATGCCAGATGAGAATCA-------  GCA-CTGAAAATGCCAGATGAGGCTCACNCCAAC  GCACCTGAAAATGCC-------------------  GCA-CTGAAAATGCCAGATGAGGCT---------  GCA-CTGAAAATGCC-------------------  ---------------------------------- | 1152 |  |

**Table 2. Result of BLAST analysis**

| Sample | Description | Max score | Total score | Query cover | E value | Ident | Accession |
| --- | --- | --- | --- | --- | --- | --- | --- |
| CnD22166 | *Cervus nippon* isolate NIP1 tRNA-Thr gene, partial sequence; tRNA-Pro gene, D-loop, and tRNA-Phe gene, complete sequence; and 12S ribosomal RNA gene, partial sequence; mitochondrial | 2058 | 2058 | 99% | 0 | 99% | KF141944.1 |
|  | *Cervus nippon* isolate NIP2 tRNA-Thr gene, partial sequence; tRNA-Pro gene, D-loop, and tRNA-Phe gene, complete sequence; and 12S ribosomal RNA gene, partial sequence; mitochondrial | 2002 | 2002 | 99% | 0 | 99% | KF141945.1 |
|  | *Cervus nippon hortulorum* mitochondrion, complete genome | 1971 | 2057 | 99% | 0 | 99% | HQ191428.1 |
|  | *Cervus nippon hortulorum* mitochondrion, complete genome | 1971 | 2057 | 99% | 0 | 99% | GU457433.1 |
| CnD22119 | *Cervus nippon* isolate NIP1 tRNA-Thr gene, partial sequence; tRNA-Pro gene, D-loop, and tRNA-Phe gene, complete sequence; and 12S ribosomal RNA gene, partial sequence; mitochondrial | 1847 | 1847 | 98% | 0 | 96% | KF141944.1 |
|  | *Cervus nippon* isolate NIP2 tRNA-Thr gene, partial sequence; tRNA-Pro gene, D-loop, and tRNA-Phe gene, complete sequence; and 12S ribosomal RNA gene, partial sequence; mitochondrial | 1825 | 1825 | 98% | 0 | 96% | KF141945.1 |
|  | *Cervus hortulorum* isolate J35D control region, partial sequence; mitochondrial | 1825 | 1825 | 87% | 0 | 99% | JF893529.1 |
|  | *Cervus nippon kopschi* mitochondrion, complete genome | 1820 | 1906 | 98% | 0 | 97% | JN389444.1 |
| CnD11102 | *Cervus nippon* isolate NIP1 tRNA-Thr gene, partial sequence; tRNA-Pro gene, D-loop, and tRNA-Phe gene, complete sequence; and 12S ribosomal RNA gene, partial sequence; mitochondrial | 2034 | 2034 | 99% | 0 | 99% | KF141944.1 |
|  | *Cervus nippon* isolate NIP2 tRNA-Thr gene, partial sequence; tRNA-Pro gene, D-loop, and tRNA-Phe gene, complete sequence; and 12S ribosomal RNA gene, partial sequence; mitochondrial | 1978 | 1978 | 99% | 0 | 99% | KF141945.1 |
|  | *Cervus nippon hortulorum* mitochondrion, complete genome | 1960 | 2033 | 99% | 0 | 99% | HQ191428.1 |
|  | *Cervus nippon hortulorum* mitochondrion, complete genome | 1960 | 2033 | 99% | 0 | 99% | GU457433.1 |
| CnD6 | *Cervus nippon* isolate NIP1 tRNA-Thr gene, partial sequence; tRNA-Pro gene, D-loop, and tRNA-Phe gene, complete sequence; and 12S ribosomal RNA gene, partial sequence; mitochondrial | 1847 | 1847 | 99% | 0 | 96% | KF141944.1 |
|  | *Cervus hortulorum* isolate J35D control region, partial sequence; mitochondrial | 1831 | 1831 | 88% | 0 | 100% | JF893529.1 |
|  | *Cervus nippon* isolate NIP2 tRNA-Thr gene, partial sequence; tRNA-Pro gene, D-loop, and tRNA-Phe gene, complete sequence; and 12S ribosomal RNA gene, partial sequence; mitochondrial | 1825 | 1825 | 99% | 0 | 96% | KF141945.1 |
|  | *Cervus hortulorum* isolate J129D control region, partial sequence; mitochondrial | 1825 | 1825 | 88% | 0 | 99% | JF893535.1 |
| CnD8 | *Cervus nippon* isolate NIP1 tRNA-Thr gene, partial sequence; tRNA-Pro gene, D-loop, and tRNA-Phe gene, complete sequence; and 12S ribosomal RNA gene, partial sequence; mitochondrial | 1845 | 1845 | 99% | 0 | 97% | KF141944.1 |
|  | *Cervus hortulorum* isolate J35D control region, partial sequence; mitochondrial | 1831 | 1831 | 89% | 0 | 100% | JF893529.1 |
|  | *Cervus nippon kopschi* mitochondrion, complete genome | 1825 | 1904 | 99% | 0 | 97% | JN389444.1 |
|  | *Cervus nippon sichuanicus* mitochondrion, complete genome | 1825 | 1904 | 99% | 0 | 97% | JN389443.1 |
| WCnD | *Cervus nippon taiouanus* mitochondrion, complete genome | 1954 | 1954 | 97% | 0 | 99% | EF058308.1 |
|  | *Cervus nippon taiouanus* mitochondrial DNA, D-loop region and tRNA-Phe, partial sequence, haplotype: 4Twn1 | 1868 | 1868 | 92% | 0 | 99% | AB279722.1 |
|  | *Cervus nippon* isolate NIP1 tRNA-Thr gene, partial sequence; tRNA-Pro gene, D-loop, and tRNA-Phe gene, complete sequence; and 12S ribosomal RNA gene, partial sequence; mitochondrial | 1855 | 1855 | 99% | 0 | 97% | KF141944.1 |
|  | *Cervus nippon sichuanicus* mitochondrion, complete genome | 1845 | 1845 | 97% | 0 | 98% | JN389443.1 |

**Table 3. The embryo development rate of SCNT in porcine oocytes**

| No. of oocytes used | No. of embryos cleaved | Type of Medium | Percentage (%)^a^ of embryos that developed to | | | | |
| --- | --- | --- | --- | --- | --- | --- | --- |
|  |  |  | 2-cell | 4-cell | 8-cell | Morula | Blastocyst |
| 110 | 30 | NCSU-23 | 25.4±0.6 | 13.6±0.6^*^ | 12.4±0.4 | 11.8±0.3 | 7.9±0.2^*^ |
| 120 | 30 | PZM-3 | 22.4±0.5 | 11.6±0.4 | - | - | - |
| 110 | 30 | PZM-5 | 26±0.5^*^ | 13±0.3^*^ | 19.4±0.2^*^ | 11.4±0.2 | 6.8±0.1 |

^a^ Percentage of embryos cultured.

^*^ Different letters within the same column represent significant differences (*p* < 0.05).

**Table 4. The embryo development rate of SCNT in porcine oocytes with co-culture**

| No. of oocytes used | No. of cleaved embryos | Percentage (%)^a^ of embryos that developed to | | | | | | |
| --- | --- | --- | --- | --- | --- | --- | --- | --- |
|  |  | Co-culture system instead of cell type | | 2-cell | 4-cell | 8-cell | Morula | Blastocyst |
| 110 | 30 | CC | - | 23.9±0.3 | 11.4±0.3 | 9.0±0.4 | 8.4±0.7 | - |
| 110 | 30 | OEC | - | 23.6±0.6 | 13.9±0.6 | 9.6±0.6 | 7.9±0.5 | - |
| 120 | 30 | UEC | - | 22.8±1.8 | 12.4±0.7 | 11.2±0.6 | 9.6±0.4 | 8.2±0.4 |
| 110 | 30 |  | LH | 13.9±0.6 | 12.7±0.6 | 8.5±0.2 | 6.9±0.7 | - |
| 120 | 30 |  | P4 | 26.0±0.7* | 23.5±0.5* | 13.0±0.7* | 12.4±0.8* | 12.1±0.4* |
| 120 | 30 |  | GTH | 23.3±0.7 | 22.7±0.4 | 10.2±0.8 | 7.8±0.5 | - |

^a^ Percentage of embryos cultured.

* Different letters within the same column represent significant differences (*p* < 0.05).

CC : Cumulus cell.

OEC : Oviduct epithelial cell.

UEC : Uterine endometrium cell.
