# Supplementary material for: iSCNT embryo culture system for restoration of Cervus nippon hortulorum, presumed to be sika deer in the Korean Peninsula
Source: PLoS One. 2024 Apr 18;19(4):e0300754. doi: 10.1371/journal.pone.0300754 (PMC11025863; doi:10.1371/journal.pone.0300754)
Supplement: Attachment — Submitted filename: 2rounds_Response to Reviewers.docx [file pone.0300754.s002.docx]

December 18, 2023

Re: MS No.

PONE-D-22-34204

Dear. Czech Journal of Animal Science editor.

Thank you for reviewing the manuscript by Lee et al., “**iSCNT embryo culture system for restoration of Cervus nippon hortulorum, presumed to be sika deer in the Korean Peninsula** (submitted as PONE-D-22-34204)”. We very much appreciate the opportunity to submit a revised version for publication. We tried to meet the reviewers' suggestions by re-phrasing part of the text as well as by quoting references. Point-by-point answers to reviewers' suggestions and questions are also listed. We hope that now the revised manuscript is suitable for the publication in your journal.

Sincerely,

Yong-Su Park, Min-Gee Oh, Sang Hwan Kim

School of Animal Life Convergence Science,

Hankyong National University,

Anseong Gyeonggi-do 17579,

Republic of Korea

(Tel: 82-10-2387-0583, Fax: 82-31-670-5127, E-mail: immunoking@hknu.ac.kr)

**Revision Note**

▶ All specified items have been modified and marked in red.

**Comments from the Editors and Reviewers:**

**Editor:**

We've checked your submission and before we can proceed, we need you to address the following issues:

1. We notice that your manuscript file was uploaded on December 26 2022. Please can you upload the latest version of your revised manuscript as the main article file, ensuring that does not contain any tracked changes or highlighting. This will be used in the production process if your manuscript is accepted. Please follow this link for more information: http://blogs.PLOS.org/everyone/2011/05/10/how-to-submit-your-revised-manuscript/

▶ We checked by referring to the link.

2. Thank you for updating your data availability statement. You note that your data are available within the Supporting Information files, but no such files have been included with your submission. At this time we ask that you please upload your minimal data set as a Supporting Information file, or to a public repository such as Figshare or Dryad.

Please also ensure that when you upload your file you include separate captions for your supplementary files at the end of your manuscript.

As soon as you confirm the location of the data underlying your findings, we will be able to proceed with the review of your submission.

▶ Has confirmed. thank you.

3. Please include a figure label and title for Figures 1, 2, 3A, 3B, 4 in your main manuscript.

▶ Both tables and figures were added to the manuscript.

4. Please include your tables as part of your main manuscript and remove the individual files. Please note that supplementary tables (should remain/ be uploaded) as separate "Supporting Information" files

▶ Removed.

5. Thank you for stating the following financial disclosure:

"The funders had no role in study design, data collection and analysis, decision to publish, or preparation of the manuscript."

At this time, please address the following queries:

a) Please clarify the sources of funding (financial or material support) for your study. List the grants or organizations that supported your study, including funding received from your institution.

b) State what role the funders took in the study. If the funders had no role in your study, please state: “The funders had no role in study design, data collection and analysis, decision to publish, or preparation of the manuscript.”

c) If any authors received a salary from any of your funders, please state which authors and which funders.

d) If you did not receive any funding for this study, please state: “The authors received no specific funding for this work.”

Please include your amended statements within your cover letter; we will change the online submission form on your behalf.

▶ I will double-check what you said.

6. Thank you for stating the following in your Competing Interests section: "NO authors have competing interests"

Please complete your Competing Interests on the online submission form to state any Competing Interests. If you have no competing interests, please state ""The authors have declared that no competing interests exist."", as detailed online in our guide for authors at http://journals.plos.org/plosone/s/submit-now

This information should be included in your cover letter; we will change the online submission form on your behalf.

▶ I will double-check what you said.

7. Can you please upload an additional copy of your revised manuscript that does not contain any tracked changes or highlighting as your main article file. This will be used in the production process if your manuscript is accepted. Please amend the file type for the file showing your changes to Revised Manuscript w/tracked changes. Please follow this link for more information: http://blogs.PLOS.org/everyone/2011/05/10/how-to-submit-your-revised-manuscript/

▶ I modified the file format name to 'Revised Article with Changes Highlighted' and uploaded it.

*Confirmation is very late as the letter did not arrive at the registered email address. Please confirm.

*I have corrected everything you mentioned.
